# Age-related reduction in motor adaptation: brain structural correlates and the role of explicit memory

**DOI:** 10.1016/j.neurobiolaging.2020.02.016

**Published:** 2020-06

**Authors:** Noham Wolpe, James N. Ingram, Kamen A. Tsvetanov, Richard N. Henson, Daniel M. Wolpert, Cam-CAN, Lorraine K. Tyler, Carol Brayne, Edward T. Bullmore, Andrew C. Calder, Rhodri Cusack, Tim Dalgleish, John Duncan, Fiona E. Matthews, William D. Marslen-Wilson, Meredith A. Shafto, Karen Campbell, Teresa Cheung, Simon Davis, Linda Geerligs, Rogier Kievit, Anna McCarrey, Abdur Mustafa, Darren Price, David Samu, Jason R. Taylor, Matthias Treder, Janna van Belle, Nitin Williams, Lauren Bates, Tina Emery, Sharon Erzinçlioglu, Andrew Gadie, Sofia Gerbase, Stanimira Georgieva, Claire Hanley, Beth Parkin, David Troy, Tibor Auer, Marta Correia, Lu Gao, Emma Green, Rafael Henriques, Jodie Allen, Gillian Amery, Liana Amunts, Anne Barcroft, Amanda Castle, Cheryl Dias, Jonathan Dowrick, Melissa Fair, Hayley Fisher, Anna Goulding, Adarsh Grewal, Geoff Hale, Andrew Hilton, Frances Johnson, Patricia Johnston, Thea Kavanagh-Williamson, Magdalena Kwasniewska, Alison McMinn, Kim Norman, Jessica Penrose, Fiona Roby, Diane Rowland, John Sargeant, Maggie Squire, Beth Stevens, Aldabra Stoddart, Cheryl Stone, Tracy Thompson, Ozlem Yazlik, Dan Barnes, Marie Dixon, Jaya Hillman, Joanne Mitchell, Laura Villis, James B. Rowe

**Affiliations:** f Cambridge Centre for Ageing and Neuroscience (Cam-CAN), University of Cambridge, Cambridge, UK; a Department of Clinical Neurosciences, University of Cambridge, Cambridge, UK; b Medical Research Council Cognition and Brain Sciences Unit, University of Cambridge, Cambridge, UK; c Computational and Biological Learning Laboratory, Department of Engineering, University of Cambridge, Cambridge, UK; d Zuckerman Mind Brain Behavior Institute, Department of Neuroscience, Columbia University, New York, NY, USA; e Centre for Speech, Language and the Brain, Department of Psychology, University of Cambridge, Cambridge, UK

**Keywords:** Sensorimotor adaptation, Ageing, Motor control, Explicit memory, Cerebellum, Medial temporal lobe

## Abstract

The adaption of movement to changes in the environment varies across life span. Recent evidence has linked motor adaptation and its reduction with age to differences in “explicit” learning processes. We examine differences in brain structure and cognition underlying motor adaptation in a population-based cohort (n = 322, aged 18–89 years) using a visuomotor learning task and structural magnetic resonance imaging. Reduced motor adaptation with age was associated with reduced volume in striatum, prefrontal, and sensorimotor cortical regions, but not cerebellum. Medial temporal lobe volume, including the hippocampus, became a stronger determinant of motor adaptation with age. Consistent with the role of the medial temporal lobes, declarative long-term memory showed a similar interaction, whereby memory was more positively correlated with motor adaptation with increasing age. By contrast, visual short-term memory was related to motor adaptation, independently of age. These results support the hypothesis that cerebellar learning is largely unaffected in old age, and the reduction in motor adaptation with age is driven by a decline in explicit memory systems.

## Highlights

• Reduced motor adaptation with age relates to less graey matter in motor regions.
• These regions are striatum, premotor and prefrontal cortex, but not the cerebellum.
• With age, motor adaptation is more positively related to medial temporal lobe areas.
• Motor adaptation relates to short-term memory measures regardless of age.
• With age, motor adaptation is more positively related to long-term memory scores.

## 1 Introduction

The sensorimotor system has a remarkable capacity to adapt to changes that occur both externally in the environment and internally in neuronal and musculoskeletal dynamics. Such adaptation is critical for learning new skills, and for adjusting previously learned movements in the face of new tasks (Franklin and Wolpert, 2011, Scott, 2004, Wolpert et al., 2011). For example, developmental and aging processes that occur throughout the lifespan—from changes in muscle and joint physiology to neuronal degeneration in the nervous system—require constant adaptation. However, motor adaptation itself is often impaired with age (Buch et al., 2003, Fernández-Ruiz et al., 2000, King et al., 2013, Seidler, 2007; but see Heuer and Hegele, 2008b, Roller et al., 2002). This calls for a better understanding of age-related changes in motor adaptation, to both understand healthy aging and inform effective rehabilitation strategies for older people affected by neurodegeneration or stroke.

To explain the effects of age on motor adaptation, optimal control theory proposes that during the execution of a voluntary movement, the central nervous system continuously simulates one’s interaction with the environment (for a review see Franklin and Wolpert, 2011). This may be achieved through an internal forward model, which learns to predict the sensory outcome of an action (Miall and Wolpert, 1996). An error signal between the predicted and actual sensory information leads to the update of the internal model, which facilitates better prediction and improved performance of future actions (Shadmehr et al., 2010). Updating an internal model is believed to be an implicit learning process, central to motor adaptation (Shadmehr et al., 2010, Wolpert et al., 2011). However, there is little (Trewartha et al., 2014) or no (Heuer and Hegele, 2008b, Vandevoorde and Orban de Xivry, 2019a) age-related decline in this implicit learning process. These findings have led to the suggestion that the decline in motor adaptation with age is independent of implicit learning and results instead from deterioration in explicit learning processes (Vandevoorde and Orban de Xivry, 2019a).

Although motor adaptation was once considered to be an archetype of implicit memory, an additional explicit learning process has been shown to contribute to motor adaptation (Heuer and Hegele, 2008b, Taylor and Ivry, 2011). This explicit process is proposed to be supported by high-level cognitive strategies that counteract changes in the environment (Taylor and Ivry, 2013), and is related to individual differences in spatial working memory performance (Christou et al., 2016, Langan and Seidler, 2011, Trewartha et al., 2014). The reduction in motor adaptation with age is tightly coupled to the reduction in the explicit learning component, while cerebellar-based learning mechanisms may not significantly deteriorate with age despite a degree of cerebellar degeneration (Vandevoorde and Orban de Xivry, 2019a). However, this hypothesis about the neural bases of age-related decline in motor adaptation has yet to be directly tested.

Here, we sought to examine the brain structural correlates of age-related decline in motor adaptation. Participants were recruited from a large population-derived cohort, aged 18–89 years, at the Cambridge Centre for Aging and Neuroscience (Cam-CAN; Shafto et al., 2014). Participants performed a visuomotor rotation learning task (c.f. Buch et al., 2003), in which they moved a stylus-controlled cursor to a visual target. A 30° angular rotation of visual feedback between the cursor and stylus location was then introduced, requiring participants to adapt their movement to overcome this visuomotor rotation so as to reach the target. In our main analyses, we conducted voxel-based morphometry (VBM) to look for correlations between gray matter volume and motor adaptation with age. Based on recent results suggesting a relative preservation of cerebellar-based motor adaptation in old age (Vandevoorde and Orban de Xivry, 2019a), we hypothesized that the reduced adaptation with age would not be related to gray matter volume differences in the cerebellum, despite an overall age-related reduction in this region. Instead, regions associated with explicit learning (e.g., dorsolateral prefrontal cortex for working memory; Anguera et al., 2010, Anguera et al., 2011) and declarative memory (e.g., medial temporal lobe; Hamann et al., 2014, Mary et al., 2017) would be related to the age-related reduction in adaptation. Based on the results of the structural imaging analyses, we performed additional post hoc behavioral analyses on the relationship between motor adaptation, age, and such explicit memory measures.

## 2 Materials and methods

### 2.1 Participants

Participants took part in the second stage of the Cam-CAN (Shafto et al., 2014). A full list of exclusion criteria is described in Table 1 in Shafto et al. (2014), including significant cognitive impairment (mini mental state examination score lower than 24), communication difficulties, significant medical problems (full list in Table 1 in Shafto et al., 2014), mobility problems, substance abuse, and MRI/MEG safety and comfort issues.

The demographic details of the participants are summarized in Table 1. The number of participants was similar across the age deciles. Of the 322 participants who performed the visuomotor learning behavioral task, 310 participants completed MRI. The study was approved by the Cambridgeshire 2 (now East of England—Cambridge Central) Research Ethics Committee, and all participants provided a written informed consent prior to the study.

**Table 1:** Summary of participant demographics across age decades

Age	N	Sex (male/female)	Handedness (right/left)	Education^a^			
				None	GCSE	A levels	University
18–29	33	13/20	30/3	0	5	6	22
30–39	46	24/22	40/6	0	2	7	37
40–49	60	28/32	51/9	1	7	4	48
50–59	46	25/21	41/5	3	5	15	23
60–69	55	31/24	50/5	3	10	14	28
70–79	53	22/31	49/4	6	8	9	30
80–89	29	16/13	28/1	5	4	12	8
Total	322	159/163	289/33	18	41	67	196

a Categorized according to the British education system: “none” = no education over the age of 16 y; “GCSE” = General Certificate of Secondary Education; “A Levels” = General Certificate of Education Advanced Level; “University” = undergraduate or graduate degree.

### 2.2 Motor adaptation task procedure and analysis

**Fig. 1:**
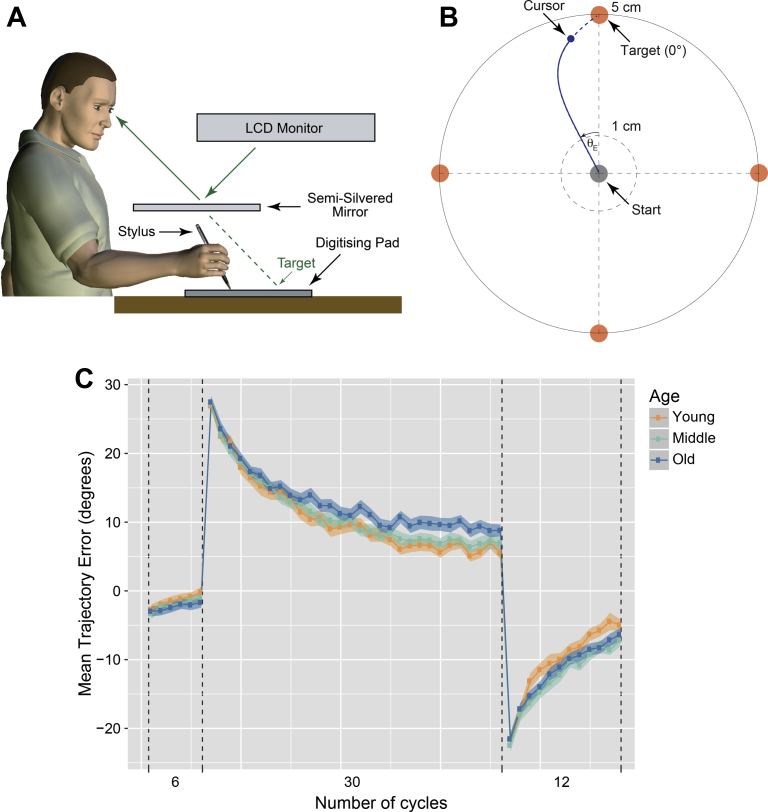
Visuomotor rotation learning task. (A) Illustration of the task in which participants moved a stylus-controlled cursor so as to hit a target. The target appeared pseudo-randomly in one of the 4 locations on the screen (once in each of the 4-trial cycles). Participants could not see their hand, and the visual feedback of the cursor was either veridical (pre-exposure and post-exposure phases) or rotated by 30° (exposure phase) relative to the stylus. (B) Participant movement adaptation was assessed by looking at the changes in their initial trajectory error θ_E_, calculated 1 cm after starting the movement. (C) Mean trajectory error across the experimental cycles (±1 standard error shaded). Dashed vertical lines separate the phases: pre-exposure (left), exposure (middle), and post-exposure (right). For illustration purposes only, data were split into 3 age groups of a similar size (“young” = 18–45 years, N = 109; “middle” = 46–65 years, N = 102; “old” = 66–89 years, N = 108), although all analyses were performed with age as a continuous variable.

Participants were asked to move a cursor so as to hit a target (Fig. 1A). To do so, they grasped a stylus pen with their dominant hand, and the position of the tip of the stylus was recorded using a digitizing touch pad (Bamboo CTH-661; Wacom Technology Corporation, Vancouver, WA) and displayed as a red cursor (radius 0.25 cm) on a computer monitor. Participants viewed the display in a semi-reflective mirror, such that the image appeared to be projected onto the horizontal surface of the touch pad. In this way, the red cursor could track the position of the stylus on the pad. The task was to move the cursor from a central “home” position (white disc radius 0.5 cm) to hit one of the 4 possible targets (yellow discs, radius 0.5 cm). Targets were displayed 5 cm from the home position and target direction was chosen from the set {0°, 90°, 180°, 270°}, in a pseudo-random order, such that each cycle of 4 consecutive trials contained each target direction. When participants successfully hit a target, it bursts and a tone was played to indicate that the trial was successful. If participants failed to initiate movement within 1 second, or to hit the target within 800 ms after movement initiation, an error tone was played and the message “Too slow” was displayed. Participants completed an initial familiarization phase of 24 trials (6 cycles of the 4 targets), during which they were permitted to see their hand and the stylus through the mirror. In the main experiment, an occluder was placed behind the mirror to prevent participants from seeing their hand.

The main experiment consisted of 192 trials which were divided into 3 phases. During the pre-exposure phase, participants performed 24 trials (6 cycles of 4 trials) in which the red cursor accurately represented the position of the stylus. During the subsequent exposure phase, participants performed 120 trials (30 cycles) in which the position of the cursor was rotated 30° clockwise relative to the central home position. The introduction of the rotation required participants to adapt their movement trajectories in order to successfully hit the targets. Finally, during the post-exposure phase, participants performed 48 trials (12 cycles) with the perturbation removed, as in the pre-exposure phase. The post-exposure phase required participants to “de-adapt” their movement trajectories in order to hit the target.

Motor adaptation on each trial was assessed by measuring the initial movement trajectory error, which is considered to reflect the feedforward component of the movement, before feedback becomes available. The trajectory error was calculated as the difference between the target angle and the angle of the initial cursor movement trajectory. The initial trajectory angle was calculated at 1 cm into the movement, relative to the start position (trials were excluded if the cursor moved less than 1 cm from the home position, affecting 0.76% of trials on average across participants). Trajectory errors were averaged across each cycle of 4 trials to give a time series across the 48 cycles (from 192 trials) of the experiment.

For each participant, trajectory errors across cycles in the exposure and post-exposure phases were each fit with an exponential function of the general form:

$$y_{i}=offset+gain\;*\;e^{\frac{x}{-\tau }}$$

where *y*_*i*_ is the trajectory error on cycle *i*, *x* is the cycle number, and τ is the exponential time constant. The fitting algorithm (“nlinfit” function in Matlab 2017a; MathWorks Inc., Natick, MA) used iteratively reweighted least squares with a bisquare weighting function. The time constants of the exponential functions for the exposure and post-exposure phases were fit as free parameters, whereas the offset and gains were constrained as follows. For the exposure phase, the trajectory error on the first cycle was constrained to be 30° (the perturbation magnitude), whereas the trajectory error on the last cycle was fitted as a free parameter (referred to as the final error). For the post-exposure phase, the trajectory error on the first cycle was constrained to be the final error (adaptation on the last cycle of the exposure phase, as described above) and the exponential had a zero asymptote.

The exponential fits therefore had 3 free parameters: (1) final adaptation (in degrees), which is the difference between the angular perturbation of 30° and the fitted final error (between 0° and 30°); (2) exponential time constant for adaptation (in cycles); and (3) de-adaptation time constant (in cycles). Based on the fit, we also calculated: (1) final de-adaptation, which is the trajectory error on the last cycle of the post-exposure phase; (2) time to half adaptation, which is the time (in cycles) to reach half the final adaptation; and (3) time to half de-adaptation (in cycles). The time constant (or time to half adaptation) is equivalent to the learning rate when the latter is defined relative to the “reducible” error, that is, based on the amount by which error is reduced during adaptation. Time to half adaptation and de-adaption was chosen over the time constants for the analyses as they were more robust across participants. Three participants (aged 28, 48, and 58 years) were excluded because their fitted final adaptation was 0°, implying failure to understand or perform the task (>5 standard deviation from cohort mean).

### 2.3 Structural neuroimaging protocol and analysis

A 3T Siemens TIM Trio with a 32-channel head coil was used to scan 310 participants (12 participants declined MRI). Both a T1-weighted MPRAGE image (repetition time 2250 ms, echo time 2.99 ms, inversion time 900 ms, field angle 9°, field-of-view 256 mm × 240 mm × 192 mm, isotropic 1 mm voxels) and a T2-weighted SPACE image (repetition time 2800 ms, echo time 408 ms, field-of-view 256 mm × 256 mm × 192 mm, isotropic 1 mm voxels) were acquired. The MR data of 8 participants were not included in the analysis due to technical problems during scanning or pre-processing problems. Together with the exclusion of 3 participants due to outlying behavioral data (see above), 299 participants were included in the structural imaging analyses.

The structural images were preprocessed for a VBM analysis, as previously described (Taylor et al., 2017) using SPM12 (www.fil.ion.ucl.ac.uk/spm) as called by the automatic analysis batching system (Cusack et al., 2015). Multimodal segmentation (using both T1- and T2-weighted images) was used to reduce age-biased tissue priors. Diffeomorphic Anatomical Registration Through Exponentiated Lie Algebra approach was applied to improve inter-participant alignment (Ashburner, 2007) as follows. Segmented images were warped to a project-specific template while modeling the shape of each brain. The resulting images were affine-transformed to the Montreal Neurological Institute space using the template and individual brain parameters. Voxel size of the normalized images was 1.5 mm isotropic. These normalization steps were followed by modulation by the Jacobean of the combined transformations (to preserve volume) and smoothing with an 8-mm full width at half maximum Gaussian kernel. A threshold of 0.15 was used on these images for the inclusion of gray matter voxels, as in previous analysis (Wolpe et al., 2016).

Multiple regressions were performed to create a statistical parametric map of differences in gray matter volume in relation to adaptation. Adaptation, age, and the (mean-corrected and orthogonalized) interaction term between adaptation and age were included as the main covariates of interest. Handedness (Edinburgh handedness score as a numerical variable), gender (categorical variable), education (categorical variables according to Table 1), mean pre-exposure trajectory error, and total intracranial volume were also included in the regression model as covariates of no interest. All variables were *z*-scored before entering the regression analyses. In addition to the positive and negative effects of adaptation and adaptation by age interaction, a conjunction analysis was performed on the combined effects of adaptation (positive effect) and age (negative effect), tested against global null hypothesis (Nichols et al., 2005), in order to identify clusters where age-related decline in gray matter volume was related to reduced adaptation. Unless stated otherwise, clusters were identified at *p* < 0.05, family-wise-error- (FWE-) corrected, with a cluster-forming threshold of *p* < 0.001, uncorrected. Significant clusters were labeled according to the Harvard-Oxford and Juelich probabilistic atlases in FSL (http://fsl.fmrib.ox.ac.uk/fsl/). Outliers in gray matter volume were identified with the robust correlation toolbox for Matlab (Pernet et al., 2013), using “detect_outliers” function and the intersection of all 3 outlier detection methods (see Pernet et al., 2013).

### 2.4 Additional behavioral analyses

To complement our structural imaging analyses, we examined the association between declarative long-term memory (LTM) performance and age-related differences in motor adaptation. As part of Cam-CAN, all participants completed the Anna Thompson Story Recall task, which is a logical memory test from the Wechsler Memory Scale Third UK edition (Weschler, 1999). In brief, participants listened to a short story and were asked to retell the story: (1) immediately after hearing it and (2) after a 30-minute delay. The story was segmented into 25 “elements,” and participants were scored according to the total number of elements recalled. For our measure of LTM performance, we used the number of elements obtained after the 30-minute delay.

In addition to LTM, 306 of the 322 participants who performed the visuomotor rotation task also completed a visual short-term memory (STM) task (Shafto et al., 2014). In brief, the STM task was a continuous color report paradigm, requiring participants to memorize and match the color of a stimulus after a short delay (Mitchell and Cusack, 2018). One to four color disks were displayed on each trial for 250 ms, followed by a blank screen displayed for 900 ms and then a probe display. In the probe display, participants were asked to report the color of the item whose location on the screen was marked by a circular gray outline. Color matching was performed using a color wheel, and our measure of interest was the angular difference between the correct color and reported color. This was summarized across trials as the root-mean-square error, collapsed across all task conditions (Mitchell and Cusack, 2018). Compared to previous studies correlating spatial working memory capacity with motor adaptation (Christou et al., 2016, Trewartha et al., 2014, Vandevoorde and Orban de Xivry, 2019a), our STM task therefore had a smaller spatial component.

The behavioral data were entered into linear regression models, in which final adaptation was the dependent variable. Separate models were run for LTM and STM. Independent variables were age, LTM or STM, and their (mean-corrected and orthogonalized) interaction. In both models, covariates of no interest were equivalent to those in the structural imaging analyses, and included mean trajectory error during the pre-exposure phase (accounting for individual movement bias, e.g., see Buch et al., 2003), education (categories according to Table 1), gender (categorical variable), and handedness (Edinburgh Handedness Score as a numerical variable; Oldfield, 1971). All variables were *z*-scored before entering the regression analysis. Multiple regressions were performed as a path model using the Lavaan package (Rosseel, 2012) in R (R Core Team, 2016), using Full Information Maximum Likelihood to account for missing data.

All statistical analyses were performed with a two-tailed alpha threshold of 0.05, but given the large sample size, we focus on effect size, here reported as the percentage of variance explained by the specific statistical contrast (*R*^2^ values more than ∼0.012 correspond to a two-tailed *p* < 0.05). For the regression analyses, we report the standardized coefficients. Plots were generated using ggplot2 (Wickham, 2009). All the raw data are available (request via http://www.mrc-cbu.cam.ac.uk/datasets/camcan/). Analysis code for this study is available on https://osf.io/v9gwj/.

## 3 Results

### 3.1 Differences in motor adaptation with age

For each participant, we examined the initial movement trajectory error (Fig. 1B) in each cycle across the 3 experimental phases. Although age was modeled as a continuous variable in all the following analyses, for ease of visualization, Fig. 1C illustrates participants’ trajectory errors for the cohort divided by age into 3 groups of similar size. During the pre-exposure phase, there was a small but consistent counter clockwise (negative angle) bias in trajectory errors across participants (absolute mean bias across all pre-exposure cycles less than 2°; *t*_(318)_ = −11.793, *p* = 7.116 × 10^−27^, *R*^2^ = 0.304). In view of a trend for a correlation of this bias with age (*r*_(317)_ = −0.108, *p* = 0.054, *R*^2^ = 0.012), we adjusted for individual differences in pre-exposure error in line with previous studies (Buch et al., 2003).

**Fig. 2:**
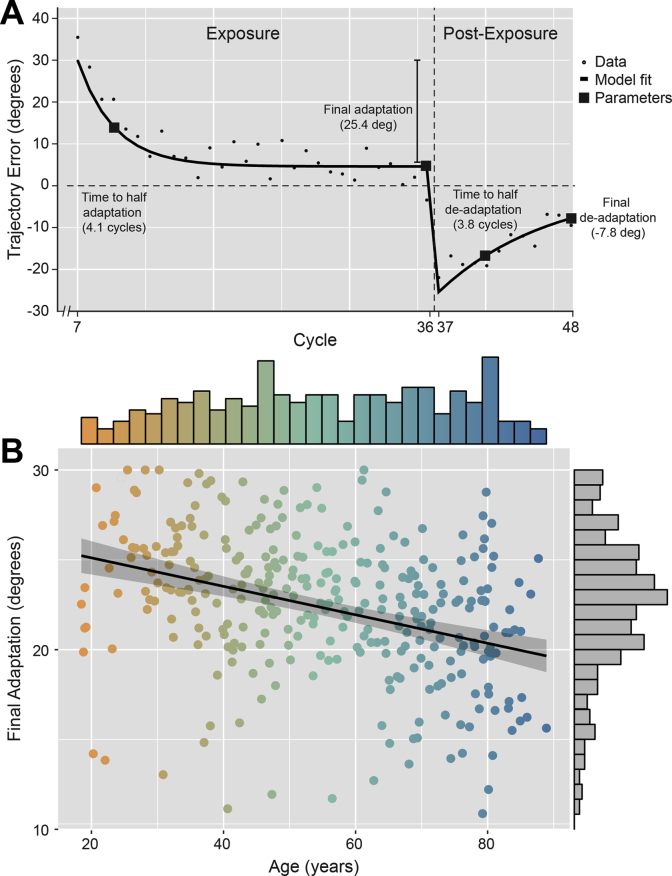
Final adaptation across age. (A) Example of the model fit in a representative participant. The model consisted of 2 sequential exponential curves, fit with a robust bisquare weight function. The main parameter of interest was “final adaptation.” (B) Correlation between final adaptation and age (with marginal histograms). Solid line indicates the linear regression fit with 95% confidence interval (gray shade).

In the exposure and post-exposure phases, participants gradually adapted their initial movement to the onset and offset of the 30° angular rotation (Fig. 1C). For the exposure and post-exposure phases, we fit the trajectory errors of each participant with a model of separate exponential curves (Fig. 2A). The key parameter to assess learning was “final adaptation,” that is, the difference between the 30° angular perturbation and fit trajectory error on the last cycle of the exposure phase (maximum value of 30 indicates full adaptation). Additional parameters were “time to half adaptation,” that is, the time (in cycles) to reach half the final adaptation, and “final de-adaptation” and “time to half de-adaptation” for the post-exposure phase. Across participants, the model fits the data well, with a mean *R*^*2*^ of 0.742 (standard deviation = 0.177), with the model fit not differing significantly with age (*r*_(317)_ = −0.100, *p* = 0.076, *R*^2^ = 0.010).

The magnitude of final adaptation is plotted against age in Fig. 2B. We fit the association between final adaptation and age with a linear model (the Bayesian Information Criteria difference relative to a second-order polynomial model was 2.67 in favor of the linear model). There was a significant negative correlation between age and final adaptation (*r*_(317)_ = −0.349, *p* = 1.353 × 10^−10^, *R*^2^ = 0.122), suggesting that older adults adapted their initial movement trajectory less than young adults. Examining the time course of individual adaptation, there was a small correlation between “time to half adaptation” and age (*r*_(317)_ = −0.1371, *p* = 0.0143, *R*^2^ = 0.019). Despite the statistical significance, we note the small effect size.

In the post-exposure phase, participants “de-adapted” to some degree, but remained biased in the opposite direction to the experimental perturbation (Fig. 1C). Older adults de-adapted less than young adults, with a significant negative correlation between age and final de-adaptation (partial correlation with final adaptation covaried; *r*_(316)_ = −0.23, *p* = 3.50 × 10^−5^, *R*^2^ = 0.053). The time course for de-adaptation did not vary with age (*r*_(317)_ = −0.083, *p* = 0.138, *R*^2^ = 0.007). For the next analyses, as our measure of motor adaptation, we focused on final adaptation which also showed a larger effect of age.

### 3.2 Gray matter differences and reduced adaptation with age

We performed spatially unbiased, whole-brain VBM analyses of gray matter volume to examine the structural correlates of motor adaptation with age. Specifically, we were interested in brain regions that showed a relationship between gray matter and both age and adaptation, as well as brain regions where the relationship with adaptation was moderated by age.

**Fig. 3:**
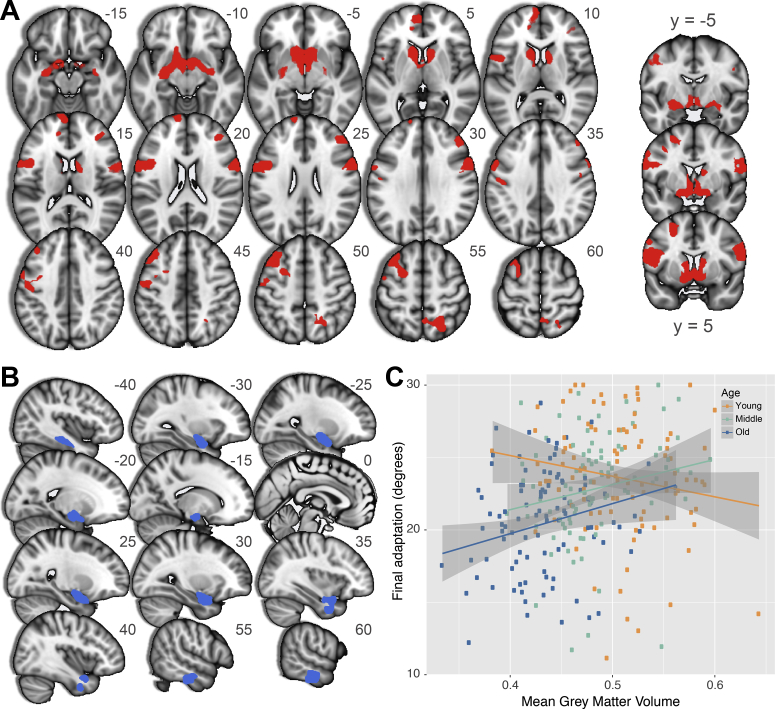
Structural imaging results. (A) Axial sections (left; numbers indicating *z* coordinate) and 2 coronal sections (right; with *y* coordinate), showing significant clusters (red) where there was a significant (*p* < 0.05, FWE-corrected) conjunction between the positive association with adaptation and negative association with age. These clusters included the striatum, bilateral premotor cortex, superior parietal lobule, and lateral frontal cortex. No such effect was found in the cerebellum. (B) Sagittal sections (numbers indicating *x* coordinate), showing 3 significant clusters (blue) where there was a significant (*p* < 0.05, FWE-corrected) positive interaction between final adaptation and age in relation to gray matter volume. These clusters included 1 in the right middle and inferior temporal lobe, and 2 clusters in the medial temporal lobes, 1 on the left and 1 on the right, each encompassing the hippocampus and amygdala. (C) Illustration of the positive interaction from (B). The interaction in all clusters was driven by a more positive relationship between gray matter volume and final adaptation in older adults than in younger participants. Mean gray matter volume extracted for left medial temporal lobe cluster for illustration of interaction direction. Groups split by age as in Fig. 1 for illustration purposes only. Abbreviation: FWE, family-wise-error.

We first examined where age-related reduction in gray matter volume was also related to reduced adaption, by testing for a conjunction between the positive association with adaptation and negative association with age. Significant clusters that survived family-wise error correction were found in the striatum (*R*^2^ = 0.064), right (*R*^2^ = 0.041) and left (*R*^2^ = 0.03) premotor cortex, left frontopolar cortex (*R*^2^ = 0.038), right superior parietal lobule (*R*^2^ = 0.035), and right ventrolateral prefrontal cortex (*R*^2^ = 0.034) extending to dorsolateral prefrontal cortex (Fig. 3A). There was no cluster showing this conjunction effect in the cerebellum, even when thresholding the conjunction at the more lenient threshold of *p* < 0.001, uncorrected, and despite age-related reduction in gray matter volume in this region (mean *T*-value of negative age correlation in cerebellar voxels = −5.1, *R*^2^ = 0.082).

Next, we identified brain areas where gray matter volume was differentially correlated with adaptation across age, by examining the interaction between final adaptation and age. No significant negative interaction was found, whereby the relationship between gray matter and adaptation decreased with age. In contrast, there was a significant positive interaction in 3 clusters: 1 encompassing the right middle and inferior temporal lobe (*R*^2^ = 0.074) and 2 clusters in the medial temporal lobes, 1 on the left (*R*^2^ = 0.063) and 1 on the right (*R*^2^ = 0.062), each encompassing the hippocampus and amygdala (Fig. 3B).

In these clusters, 3 participants whose gray matter was highest were identified as outliers (see Materials and methods section). After removing these 3 cases, the interaction remained significant in all 3 clusters at *p* < 0.001, uncorrected, and the left medial temporal lobe cluster survived FWE-correction. When split by age groups for visualization, the interaction in all 3 clusters was driven by a more positive relationship between gray matter volume and final adaptation in older than younger participants (illustrated in Fig. 3C for the left medial temporal lobe).

Given the central role of the hippocampus and prefrontal cortex in explicit learning, these results support the hypothesis that age-related decline in motor adaptation is associated with differences in explicit memory (Vandevoorde and Orban de Xivry, 2019a). We explored this in additional post hoc behavioral analyses.

### 3.3 Behavioral analyses

**Fig. 4:**
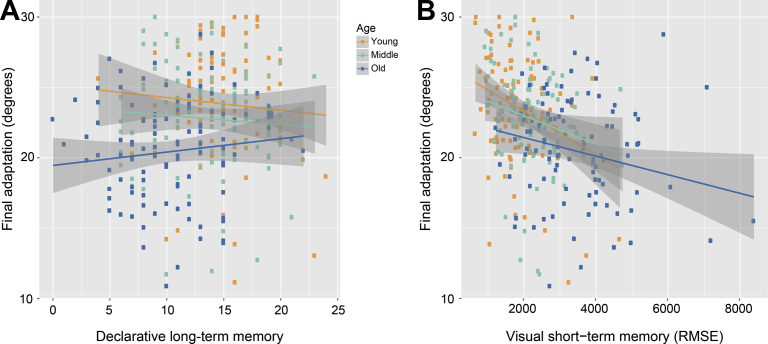
Explicit memory performance and motor adaptation by age. (A) Illustration of the positive interaction between age and declarative LTM performance in the Story Recall task in relation to final adaptation. LTM scores were the total number of items recalled after a delay period, such that higher values indicate better LTM. Groups split by age as in Fig. 1 for illustration purposes only. Solid line indicates the linear regression fit with 95% confidence interval (gray shade). (B) As in (A), but for short-term memory score in the visual STM task. STM scores were the RMSE of the difference between target and reported color, such that higher values indicate worse STM performance. Abbreviations: LTM, long-term memory; RMSE, root-mean-square error; STM, short-term memory.

To complement our structural imaging analyses, we performed post hoc behavioral analyses looking at the relationship between motor adaptation and Cam-CAN’s behavioral measures of explicit memory collected on the same cohort, namely visual STM and declarative LTM. Using a similar regression model as in the VBM analysis (i.e., adjusting for covariates of no interest), the full results are shown in Table 2 and illustrated in Fig. 4.

**Table 2:** Summary of multiple regression analysis for predicting final adaptation

	Declarative long-term memory Final adaptation			
	β Estimate	β SE	*z*-value	*p*-value
Education	0.142	0.053	2.682	0.007
Gender	0.166	0.102	1.618	0.106
Handedness	0.173	0.051	3.377	0.001
Pre-exposure bias	−0.132	0.051	−2.564	0.01
**Age**	**−0.386**	**0.058**	**−6.645**	**<0.001**
**Declarative memory**	**−0.065**	**0.060**	**−1.072**	**0.284**
**Age×declarative memory**	**0.107**	**0.052**	**2.039**	**0.041**
	Visual short-term memory Final adaptation			
	β Estimate	β SE	*z*-value	*p*-value
Education	0.115	0.052	2.215	0.027
Gender	0.07	0.103	0.682	0.495
Handedness	0.169	0.051	3.303	0.001
Pre-exposure bias	−0.144	0.051	−2.824	0.005
**Age**	**−0.199**	**0.063**	**−3.156**	**0.002**
**STM error**	**−0.213**	**0.063**	**−3.365**	**0.001**
**Age×STM error**	**0.021**	**0.051**	**0.412**	**0.680**

Regression models with declarative LTM measured as number of elements (out of 25) remembered in the Story Recall task after 30-min delay (*R*^2^ = 0.208), and STM measured as the error between the reported and target colors (*R*^2^ = 0.221). Education = categorical variable, as in Table 1; Gender = categorical variable of male (coded as 0) and female (coded as 1); Handedness = Edinburgh Handedness Score (Oldfield, 1971); Pre-exposure bias = mean trajectory error in first 6 pre-exposure cycles. Beta coefficients are standardized. Covariates of interest are in bold.

Key: LTM, long-term memory; SE, mean standard error; STM, short-term memory.

For LTM (model *R*^2^ = 0.208), performance showed no main effect on adaptation (beta_standardized_ = −0.065, *Z* = −1.072, *p* = 0.284), but its effect did interact with age (beta_standardized_ = +0.107, *Z* = 2.039, *p* = 0.041; Fig. 4A). This pattern resembled that for the VBM analyses above. For STM (model *R*^2^ = 0.221), root-mean-square of performance error had a negative effect on adaptation (over and above age) (beta_standardized_ = −0.213, *Z* = −3.365, *p* = 0.001), but this effect did not interact with age (beta_standardized_ = +0.021, *Z* = 0.412, *p* = 0.68). This suggests that worse STM performance (increased error) is related to reduced motor adaptation, regardless of age (Fig 4B). Taken together, these behavioral correlations with explicit memory measures suggest that STM capacity is generally related to motor adaptation, but that the age-related reductions in motor adaptation are related to LTM.

For completeness, we also related motor adaptation to several other cognitive variables collected in Cam-CAN (Shafto et al., 2014), as shown in the Supplementary Material. These results revealed (1) similar results when using a different measure of LTM, that is, no main effect, but a positive age × LTM interaction; (2) an association between fluid intelligence and motor adaptation (over and above age), but no age × fluid intelligence interaction, as in the STM results above; (3) no association of motor adaptation with sensory attenuation. These results further emphasize that while motor adaptation is related to explicit learning mechanisms across the lifespan, age-related changes are specifically related to declarative LTM capacity.

## 4 Discussion

These results from a large population-based cross-sectional cohort suggest that reduced sensorimotor adaptation in older adults is partially explained by age-related decline in explicit memory systems. We found that reduction in gray matter volume in the striatum and prefrontal cortex, but not in the cerebellum, was related to adaptation differences with age. Differences in the medial temporal lobe, including in the hippocampus, became more strongly associated with motor adaptation with age. These results support the hypothesis of sensorimotor adaptation as a composite of multiple learning strategies (Huberdeau et al., 2015, McDougle et al., 2016), which are differentially affected by age (Vandevoorde and Orban de Xivry, 2019a).

### 4.1 Age-related differences in sensorimotor adaptation

The degree of motor adaptation is typically reduced with age (Buch et al., 2003, Fernández-Ruiz et al., 2000, King et al., 2013, Seidler, 2007), although visuomotor adaptation is not always found to change with age (Heuer and Hegele, 2008a, Roller et al., 2002). Divergent results across studies call for careful consideration of methodological differences, such as the sample size, the magnitude of experimental perturbation, task difficulty, and type of perturbation paradigm. For example, age-related differences are more varied in smaller perturbations and easier adaptation tasks (Heuer and Hegele, 2008b), and in a force field paradigm (Trewartha et al., 2014). Large-scale studies can help reduce type II errors (false negatives), by increasing statistical power.

Across 319 participants, in a population-based study (Shafto et al., 2014), we observed a large variability in motor adaptation in adults (Fig. 2B), which may explain the contrasting findings on adaptation levels with age reported in smaller studies. The degree of motor adaptation was reduced with age with *r* = 0.35, that is, ∼12% of the variability in adaptation explained by age. An empirical investigation of individual difference studies has demonstrated that correlations with *r* = 0.3 correspond to the upper 25th percentile of effect sizes, and thus considered “large” (Gignac and Szodorai, 2016). It is likely that effects reported in previous studies were either different as a result of experimental procedures (see above), or inflated due to small sample size and publication bias (Gelman and Carlin, 2014).

The inconsistency in age-related differences in motor adaptation is supplemented by discrepancies in the reported effect of age on adaptation rate (e.g., see Fernández-Ruiz et al., 2000, Hardwick and Celnik, 2014, Trewartha et al., 2014). We found a negative correlation, albeit with a weak effect size (*r* = −0.14), between age and adaptation rate, such that older adults reached half their total amount of adaptation faster than young adults. The small effect size suggests that adaptation rate was even more highly variable across participants.

### 4.2 Age-related reduction in gray matter volume and motor adaptation

In the classical interpretation of sensorimotor adaptation, an internal forward model predicts the sensory outcome of one’s movement (Shadmehr et al., 2010, Wolpert and Flanagan, 2010). A discrepancy between sensorimotor prediction and sensory feedback (sensory prediction error) enables the internal model to be updated. This implicit learning process has been mapped to the cerebellum in a myriad of lesional, structural, and functional imaging studies (Doyon and Benali, 2005, Galea et al., 2011, Thach, 1996, Tomassini et al., 2011, Tseng et al., 2007). In recent years, however, evidence has emerged for the significant contribution of an explicit learning component (Taylor and Ivry, 2012), which is sensitive to the distance between the target and sensory feedback (namely, performance error) (Taylor et al., 2014). Individual differences in working memory capacity in young (Anguera et al., 2010) and older adults (Langan and Seidler, 2011, McNay and Willingham, 1998, Uresti-Cabrera et al., 2015) have been linked with motor adaptation in general, and the explicit component in particular (Christou et al., 2016).

Accumulating evidence has indicated a relative preservation of implicit motor adaptation, but deterioration in explicit adaptation in old age. First, when an experimental visual perturbation is small and gradual, emphasizing implicit processes, older adults adapt their movement as well as young adults (Buch et al., 2003). Second, when young and old participants are matched by explicit knowledge of the perturbation, age-related differences largely dissipate (Heuer and Hegele, 2008b). Third, the contribution of implicit and explicit learning to age-related decline in motor adaptation was recently dissociated (Vandevoorde and Orban de Xivry, 2019a). These authors compared cued and uncued perturbation trials, and inferred implicit learning as the average adaptation in uncued trials, in which cognitive strategies are believed to be switched off (Morehead et al., 2015). Age-related decline in overall motor adaptation was indeed explained by a reduced explicit component, whereas the implicit component remained intact (Vandevoorde and Orban de Xivry, 2019a). This led to the proposal that “cerebellar-based mechanisms do not deteriorate with age despite cerebellar degeneration” (Vandevoorde and Orban de Xivry, 2019a). Our results, with no significant association between age-related reduction in cerebellar gray matter volume and adaptation, are consistent with this hypothesis, although of course we cannot conclude from this null result that there is no such relationship.

An alternative interpretation comes from our measure of overall motor adaptation. We used a standard visuomotor rotation task that does not formally separate implicit and explicit contributions, as such tasks were developed after data collection for this large cross-sectional study had begun (Shafto et al., 2014, Taylor et al., 2014). Studies that have separated implicit and explicit components of motor adaptation reveal that a large proportion of the variability in overall adaptation is explained by the explicit learning component (e.g., Christou et al., 2016). The implicit component, on the other hand, is only weakly negatively correlated with overall adaptation (Vandevoorde and Orban de Xivry, 2019a). This should be taken into account when considering the lack of correlation between age-related differences in cerebellum and adaptation.

In contrast to the cerebellar null result, we found that age-related reduction in gray matter volume in bilateral premotor and lateral prefrontal cortex was related to reduced adaptation. These clusters overlap with regions that have been suggested to mediate spatial working memory capacity important for motor adaptation (Anguera et al., 2010). Functional age-related deficits in these regions are proposed to contribute to reduced motor adaptation (Anguera et al., 2011). These imaging results converge with recent behavioral findings, showing that reduced explicit motor adaptation with age may be mediated by age-related decline in spatial working memory (Vandevoorde and Orban de Xivry, 2019b).

Similarly, reduced gray matter volume with age in the striatum was related to reduced adaptation. In the context of motor adaptation, striatal plasticity is crucial for the acquisition of motor skills in animal models (Dang et al., 2006). In humans, striatal activation has been demonstrated during a motor adaptation task using functional MRI (Seidler et al., 2006), and is often implicated in tasks involving motor sequence learning (reviewed in Doyon et al., 2009). The role of the striatum, and more broadly the basal ganglia, is proposed to complement cerebellar-based (sensory prediction error-based) learning, by reinforcing movements that lead to rewarding outcomes (Bostan and Strick, 2018, Wickens et al., 2003), thereby contributing to overall adaptation (Huang et al., 2011). However, this learning strategy is known to be generally impaired in old age (Eppinger et al., 2011), and its impairment is indeed suggested to contribute to reduced motor adaptation with age (Heuer and Hegele, 2014).

### 4.3 Reduced motor adaptation and explicit memory system

Motor adaptation has been related to individual differences in explicit memory, such as performance in visuospatial working memory (Anguera et al., 2010, Christou et al., 2016, Vandevoorde and Orban de Xivry, 2019a). Vandevoorde and Orban de Xivry found a correlation between adaptation and spatial working memory for young and older adults, which related to the explicit motor adaptation component (Vandevoorde and Orban de Xivry, 2019b). Moreover, Trewartha et al. (2014) reported that better performance in a paired associate learning task was related to larger retention in the fast motor adaptation process, specifically in older adults. Interestingly, the paired-associate learning task required participants to memorize and recall stimuli in learning and test phases, which is conventionally regarded as a measure of LTM rather than STM/working memory (Trewartha et al., 2014).

Our results are consistent with these findings. Across the entire cohort, we found associations between motor adaptation with STM. Although our visual STM task did not include an explicit spatial component like the previous studies described above (Anguera et al., 2010, Christou et al., 2016, Vandevoorde and Orban de Xivry, 2019a), better STM capacity was related to motor adaptation levels for the whole cohort, including both young and older adults, over and above age. Moreover, a similar pattern of results was found for the association between motor adaptation and fluid intelligence, which generally correlates with STM/working memory (Conway et al., 2003).

We found an age-dependent link between age-related differences in motor adaptation and the brain regions associated with explicit/declarative LTM, in which (1) gray matter volume in regions of the medial temporal lobe, including the hippocampus, was positively associated with adaptation as people grow older and (2) post hoc analyses on behavioral measures of LTM showed a similar pattern to the neuroimaging results. Nonetheless, despite the convergence with the structural imaging results, we acknowledge the small effect size and exploratory nature of these correlations.

The increasingly stronger association between motor adaptation and LTM with increasing age has at least 2 interpretations. First, this interaction could reflect growing individual differences in LTM with age, in which a subset develops impaired LTM and motor adaptation, as suggested by Trewartha et al. (2014). Indeed, it is possible that the positive relationship between LTM and motor adaptation is only revealed in old age once LTM falls below a certain critical level, at which implicit learning can no longer compensate (c.f. Vandevoorde and Orban de Xivry, 2019a). Second, the interaction could reflect an increased dependence on an explicit learning mechanism for motor adaptation as age increases. Increased reliance on explicit learning strategy for motor adaptation is observed in younger adults with better explicit memory, thereby optimizing adaptation capacity (Christou et al., 2016). However, considering the substantial decline in explicit memory with age (Henson et al., 2016), such increased reliance would be deleterious, and may instead reflect a broader tendency of older adults to rely more on cognitive resources for motor performance (Seidler et al., 2010), for example, as seen during normal walking (Mirelman et al., 2017). This interpretation would be further supported by our finding of a correlation between adaptation rate and age: Since explicit learning strategies have faster learning rates (McDougle et al., 2015), such a result would support the hypothesis of increased reliance on explicit strategies for adaptation.

To our surprise, we found no association between motor adaptation and sensory attenuation. We previously hypothesized that internal models would be impervious to differences between sensory prediction and feedback with age, because of reduced reliance on “noisy” sensory information, as reflected in increased sensory attenuation (Wolpe et al., 2016). However, there was no link between motor adaptation and sensory attenuation, and differences in attenuation did not explain reduced adaptation with age. This null result may suggest a true lack of association between motor adaptation and sensory attenuation, but other interpretations are possible. For example, the measure of sensory attenuation reflects the precision-dependent down-weighting of haptic and proprioceptive feedback, whereas our motor adaptation task relied heavily on visual feedback. Attenuation might therefore be related to adaptation in other tasks, with, for example, a physical force field perturbation, rather than visual perturbation.

### 4.4 Medial temporal lobe and motor adaptation

The medial temporal lobe and hippocampus contribute to motor adaptation, but the nature of this association is not fully resolved. A previous study showed that the degree of motor adaptation is related to changes in mean diffusivity of white matter within the medial temporal lobe, such that young healthy individuals who showed increased white matter integrity were able to adapt more in a visuomotor learning task (Della-Maggiore, personal communication). The hippocampal role in adaptation may also depend on sleep (Solano et al., 2019). The hippocampus contributes to the acquisition of motor sequences, through connections with higher cortical regions (Schendan et al., 2003), and may be essential for consolidating motor memories via their interactions with the cerebellum and striatum (Doyon et al., 2009). On the other hand, patients with bilateral medial temporal lobe damage are still capable of acquiring new motor skills (Corkin, 1968). Interestingly, greater hippocampal activity has been observed in old age during motor sequence learning, which is suggested to reflect a compensatory mechanism for striatal-related degradation (reviewed in King et al., 2013). The age-related effects we observe in the medial temporal lobe, together with the association between adaptation and gray matter reduction in the striatum, are consistent with this account.

The anterior part of the hippocampus, which was identified in our study, has been demonstrated to support the learning of new environmental layouts (Maguire et al., 2000). Furthermore, in tasks involving visuospatial navigation, the anterior hippocampus is proposed to encode the Euclidean distance to one’s goal (Howard et al., 2014). This goal distance signal is speculatively analogous to the performance error signal that is used to update the explicit learning component for motor adaptation (Taylor and Ivry, 2013), a component which is specifically impaired in old age (Vandevoorde and Orban de Xivry, 2019a). Similar performance error signals have been found in the adjacent amygdala (Gemba et al., 1986), which enhances learning of highly arousing or rewarding action-outcome associations (Cador et al., 1989, Fastenrath et al., 2014). The amygdala was also identified in the same analysis as the anterior hippocampus in our study. Taken together, the medial temporal, and the anterior hippocampus and amygdala within it, may contribute to the consolidation of motor memories by encoding performance error signals that are critical for the explicit component of motor adaptation. Their degeneration with age may thus make older people more prone to motor learning deficits.

Neurological disorders are common in old age, and as populations around the world are rapidly aging, there is a growing demand for effective neurorehabilitation schemes. This is particularly evident after stroke, when patients often need to re-acquire motor skills, in the face of motor learning impairments (Krakauer, 2006). Our results underscore the challenge of developing new approaches for older patients, which emphasize implicit, sensory prediction error-based learning mechanisms to leverage intact learning systems. This may explain why rehabilitation methods that minimize cognitive strategies, and instead emphasize motor imagery and action observation, have been clinically advantageous (e.g., Garrison et al., 2010).

## 5 Conclusion

Taken together, our structural imaging and behavioral data suggest that across the lifespan, motor adaptation declines with age as a result of the deteriorating explicit learning system. Although our study focused on healthy adults, it highlights the need to consider age in tailoring rehabilitation programs, and take into account different learning systems across the adult lifespan.

## CRediT authorship contribution statement

**Noham Wolpe:** Conceptualization, Formal analysis, Software, Visualization, Writing - original draft, Writing - review & editing. **James N. Ingram:** Methodology, Conceptualization, Formal analysis, Software, Validation, Writing - review & editing. **Kamen A. Tsvetanov:** Validation, Writing - review & editing. **Richard N. Henson:** Conceptualization, Validation, Writing - review & editing. **Daniel M. Wolpert:** Methodology, Conceptualization, Software, Writing - review & editing. **James B. Rowe:** Conceptualization, Funding acquisition, Project administration, Supervision, Writing - review & editing.

## Appendix A Supplementary data

Data Profile
