# Supplementary material for: Age-related reduction in motor adaptation: brain structural correlates and the role of explicit memory
Source: Neurobiol Aging. 2020 Jun;90:13–23. doi: 10.1016/j.neurobiolaging.2020.02.016 (PMC7181181; doi:10.1016/j.neurobiolaging.2020.02.016)
Supplement: Supplementary Material [file mmc2.docx]

## SUPPLEMENTARY MATERIAL

### Supplementary Analysis 1

**Association between motor adaptation and additional behavioural variables**

For completeness, we report the correlations and regression analyses of adaptation and a few behavioural measures of potential interest, collected as part of Cam-CAN (Shafto et al. 2014). We tested the link between motor adaptation, age and the following measures:

1. Declarative long-term memory (LTM), measured using an additional, independent behavioural task, collected in a smaller subset of the participants (n=120, 116 complete datasets). These participants showed a similar interaction effect in the medial temporal lobe as reported for the whole group in the main text (Supplementary Analysis 2). The task was an Emotional Memory task, which had many more trials and so potentially provides a more sensitive measure of declarative memory (Henson et al., 2016). In brief, during a study phase, participants viewed a background picture, which varied in terms of emotional valence. After 2 seconds, a picture of an object was superimposed on the background picture. Participants were instructed to imagine a story linking the two pictures for 8 seconds, after which the next trial began. Ten minutes after completing the study phase, the test phase began. A noise-corrupted object picture was presented, and participants were asked to name the object. After 1 second, the noise was removed and participants were asked indicate their confidence about whether or not this object had appeared in the study phase (“sure new”, “think new”, “think old”, “sure old”). If participants indicated “old”, they were asked to provide a verbal description of the background picture, on which the object was superimposed in the study phase, and to indicate the emotional valence of the background picture. The description was categorised by the experimenter as “incorrect”, “not providing information”, “gist correct” or “detail correct”. For our additional measure of declarative memory performance, we used the score obtained by summing up the total number of detail correct background pictures, collapsed across emotional valence.
2. Fluid intelligence, measured with Cattell Culture Fair Test (Cattell 1971; Cattell and Cattell 1973). In total, 310 participants completed both the visuomotor rotation task and Cattell. Fluid intelligence is thought to be closely related to working memory, which in turn has been linked to motor adaptation across ages using a spatial working memory task (Trewartha et al. 2014; Christou et al. 2016; Vandevoorde and Orban de Xivry 2019). We used the total Cattell score as our measure of interest.
3. Sensory attenuation measured using a force matching task (Wolpe et al. 2016). To our knowledge, an association between sensory attenuation and motor adaptation has not yet been tested. However, age-related increase in attenuation suggests increased reliance on prediction while down-weighting a noisy sensorium, which may in turn diminish sensory error-based (implicit) learning (Wolpe et al. 2016). Our measure of interest was mean force overcompensation reflecting sensory attenuation.

Multiple regression analyses were performed to test for an association between motor adaptation and these variables (over and above age; age added as a covariate), as well as their (mean-corrected, orthogonalised) interaction with age. As in the main text, education, handedness, gender and pre-exposure bias were included as additional covariates.

The regression results are reported in full in Table S1. The results showed a main effect of fluid intelligence on motor adaptation across the whole group (over and above age), with no interaction effect. By contrast, there was no main effect of LTM as measured in the Emotional Memory task, but a LTM x memory interaction. There were no effects of sensory attenuation on motor adaptation. These results are consistent with those reported in the main text. They suggest that decline in cognitive abilities, such as fluid intelligence, can explain age-related decline in motor adaptation, while the relationship between motor adaptation and declarative memory in moderated by age.

### Supplementary analysis 2

**Grey matter differences and adaptation with age in the participants who also completed the Emotional Memory task**

We performed a similar regression analysis in the subgroup of participants who had also completed the Emotional Memory task. Specifically, we examined the positive association between the interaction of age and adaptation with grey matter volume. There were no clusters that survived the threshold of 0.05 FWE-corrected, however, at a more lenient threshold of *p*<0.001, uncorrected, two clusters in the right medial temporal lobe and left hippocampus-amygdala intersection showed a positive correlation between grey matter volume and positive age x adaptation interaction. As shown in Figure S1, these two clusters overlapped with the larger clusters reported for the whole group in the main text.

### Figure S1

Positive association between grey matter volume and age by adaptation interaction in the subgroup of participants who completed the Emotional Memory task. As in the whole-group analysis, a positive association between grey matter volume and age x adaptation interaction was found in the right medial temporal lobe and left amygdala-hippocampus intersection. Clusters from the whole-group analysis are shown in blue, and overlapping voxels with the subgroup are shown in magenta.


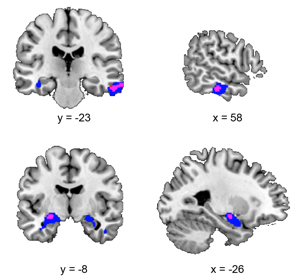


### Table S1

Regression models of final adaptation and additional cognitive measures and their interactions with age. Age, education, gender, handedness and pre-exposure bias were included as additional covariates.

|  | Final adaptation ~ | | | |
| --- | --- | --- | --- | --- |
| Variable | β estimate | β SE | z-value | p-value |
| LTM | 0.074 | 0.100 | 0.739 | 0.460 |
| LTM x Age | 0.213 | 0.082 | 2.605 | 0.009 |
| Cattell | 0.188 | 0.076 | 2.464 | 0.014 |
| Cattell x Age | 0.001 | 0.054 | 0.022 | 0.982 |
| Sensory attenuation | -0.055 | 0.053 | -1.046 | 0.296 |
| Sensory attenuation x Age | 0.038 | 0.051 | 0.749 | 0.454 |
|  |  |  |  |  |

LTM = long-term memory, measured in the Emotional Memory task. Beta estimates are standardised.

## SUPPLEMENTARY REFERENCES

Cattell RB. 1971. Abilities: Their structure, growth, and action. Boston: Houghton Mifflin.

Cattell RB, Cattell HEP. 1973. Measuring intelligence with the culture fair tests. Champaign, IL: The Institute for Personality and Ability Testing.

Christou AI, Miall RC, McNab F, Galea JM. 2016. Individual differences in explicit and implicit visuomotor learning and working memory capacity. Sci Rep. 6:1–13.

Henson RN, Campbell KL, Davis SW, Taylor JR, Emery T, Erzinclioglu S, Cam-CAN, Kievit RA. 2016. Multiple determinants of lifespan memory differences. Sci Rep. 6:32527.

Shafto MA, Tyler LK, Dixon M, Taylor JR, Rowe JB, Cusack R, Calder AJ, Marslen-Wilson WD, Duncan J, Dalgleish T, Henson RN, Brayne C, Matthews FE. 2014. The Cambridge Centre for Ageing and Neuroscience (Cam-CAN) study protocol: a cross-sectional, lifespan, multidisciplinary examination of healthy cognitive ageing. BMC Neurol. 14:204.

Trewartha KM, Garcia A, Wolpert DM, Flanagan JR. 2014. Fast but fleeting: adaptive motor learning processes associated with aging and cognitive decline. J Neurosci. 34:13411–13421.

Vandevoorde K, Orban de Xivry J-J. 2019. Internal model recalibration does not deteriorate with age while motor adaptation does. Neurobiol Aging. 80:138–153.

Wolpe N, Ingram JN, Tsvetanov KA, Geerligs L, Kievit RA, Henson RN, Wolpert DM, Cam-CAN, Rowe JB. 2016. Ageing increases reliance on sensorimotor prediction through structural and functional differences in frontostriatal circuits. Nat Commun. 7:13034.
